# COVID-19 vaccine registry for pregnant women: policy to control complications of vaccination in pregnant women in 2021–2022

**DOI:** 10.1186/s12884-023-05856-3

**Published:** 2023-07-27

**Authors:** Farkhondeh Asadi, Roya Shakiba, Reza Rabiei, Hassan Emami, Azam Sabahi

**Affiliations:** 1 Present Address: Department of Health Information Technology and Management, School of Allied Medical Sciences, Shahid Beheshti University of Medical Sciences, Tehran, Iran; 2 Department of Health Information Technology, Ferdows School of Health and Allied Medical Sciences, Birjand University of Medical Sciences, Birjand, Iran

**Keywords:** Registry, Vaccination, COVID-19 vaccines, Pregnancy

## Abstract

**Background:**

Data management related to COVID-19 vaccination in pregnant women is vital to improve the treatment process and to establish preventive programs. Implementing a registry to manage data is an essential part of this process. This study aims to design a national model of the COVID-19 vaccination registry for pregnant women in Iran.

**Methods:**

The present study is an applied descriptive study conducted in 2021 and 2022 in two stages. In the first stage, the coordinates of the National Registry of COVID-19 vaccination of pregnant women from related references and articles, as well as the comparative study of the National Registry of COVID-19 vaccination of pregnant women in the United States, Canada, and the United Kingdom was done. In the second stage, the preliminary model was designed. The model was validated using the Delphi technique and questionnaire tools and analyzing the data.

**Results:**

The presented national COVID-19 vaccination registry model of pregnant women’s main components consist of objectives, data sources, structure, minimum data set, standards, and registry processes, all of which received 100% expert consensus.

**Conclusion:**

The vaccination registry of pregnant women has a major role in managing COVID-19 vaccination data of pregnant women and can be one of the Ministry of Health and Medical Education priorities.

## Background

The COVID-19 pandemic is a global health crisis and the most significant challenge people have faced since World War II [1]. The new coronavirus (SARS-CoV-2) prevalence began in late December 2019 and spread rapidly worldwide and affected public health systems. SARS-CoV-2 causes COVID-19 respiratory disease, ranging from asymptomatic and mild upper respiratory infection symptoms to acute respiratory distress syndrome (ARDS) and death [2, 3].

Background diseases and pregnancy are associated with the risk of exacerbation of the disease in the case of COVID-19 [4]. Evidence has shown that pregnant women with COVID-19 are more susceptible to disease exacerbation than non-pregnant women in the fertility ages [5].

Pregnancy is a condition in which the mother’s immune system must overcome two critical challenges of protecting the fetus against immunological factors and dangerous pregnancy infections [6]. Due to physiological changes and factors such as higher body mass index, background disease, age over 25, lack of vaccination, and failure to observe social distancing, pregnant women are at higher risk of severe respiratory infections [7].

Complications of COVID-19 infection during pregnancy include an increased risk of preterm birth [5, 8], increased fetal transmission of infection, increased risk of preeclampsia^1^, coagulopathy^2^, the need for intensive care, and an increased risk of maternal and fetal mortality [5, 9, 10]. Certain studies also show that in infants born to women infected with COVID-19 during pregnancy, there is a possibility of the need for care in the neonatal intensive care unit (NICU) [9, 11]. Until September 27, 2021, more than 125,000 COVID-19-positive cases were confirmed by laboratory reports in pregnant women, including more than 22,000 hospitalizations and 161 deaths. The highest number of deaths from COVID-19 in pregnant women (n = 22) was reported in one month of the pandemic in August 2021. Nearly 97% of pregnant women hospitalized with confirmed SARS-CoV-2 infection in 2021, according to data from the COVID-19 hospitalized surveillance network (COVID-NET), were unvaccinated [12]. More than 218,000 people who received the COVID-19 vaccine while pregnant were found to have done so, according to the Centers for Disease Control and Prevention (CDC), which assessed the health of those who had received the vaccine by April 2022. The findings showed that no unique safety signals were seen in the pregnant trial participants, and the adverse reactions to the vaccine were similar in pregnant and non-pregnant women [13, 14].

Vaccination is the most efficient way to control the COVID-19 pandemic [15].

Vaccination is the best way to reduce the complications of SARS-CoV-2 infection in the mother and fetus. The Society for Maternal and Fetal Medicine (SMFM), the CDC, and the other organizations that monitor maternal health have recommended that pregnant women, people who are planning to become pregnant, postpartum and lactating women be provided the COVID-19 vaccine [16].

Decision-makers at all health system levels need relevant, reliable, and in-time information to improve the decision-making process. Information systems have a crucial role in generating information for managerial and operational decisions. Information systems allow decisions to be made to reduce the morbidity and mortality associated with vaccine-preventable diseases (VPDs) [17].

Vaccination immunity during pregnancy is assessed either through passive monitoring systems such as the Vaccine Adverse Event Reporting System (VAERS) and the pregnancy registry or by conducting observational studies in databases obtained from electronic health records (EHRs) [18, 19]. Registries are tools for data management, and there are different registries for managing data related to various diseases [20, 21]. The National Committee on Vital and Health Statistics (NCVHS) describes registries as an organized collection system storing, retrieving, analyzing, and publishing information about people with a particular illness. This condition that predisposes them to a health-related event, previous exposure to known substances, or conditions, or suspected adverse health effects [22]. Registries manage data through a process that includes: case finding, data collection, coding and abstracting, quality control, reporting, and patient follow-up [23].

The international registry COVID-19 Vaccines International Pregnancy Exposure (COVIPER) aims to evaluate the possible effects of single- or mixed-vaccine administration to prevent COVID-19 on obstetric, neonatal, and infant outcomes. The maternal demographic is a part of the minimum data set required for this registry.

Information, data on reproductive history, health-related behaviors, and pre-pregnancy health, the number of fetuses present, health status throughout pregnancy, concomitant medications, pregnancy outcome, date of vaccination, gestational age at the time of vaccination, number of COVID-19 vaccination doses received and whether during pregnancy, manufacturer of each COVID-19 vaccine dose, adverse events within 48 h of vaccination, start and end dates of adverse events, the outcome of adverse events, the severity of adverse events, pre-existing conditions affecting immune response, other non-COVID-19 vaccines received in four weeks were considered prior to COVID-19 vaccine. The items followed up in this registry include side effects in mother and baby, baby weight, baby gender, and admission to the NICU [24].

One of the registries in America is the V-safe COVID-19 Vaccine Pregnancy Registry, aiming to record side effects after receiving the COVID-19 vaccine. The data collected in this registry includes pregnancy outcomes, pregnancy complications, and infant outcomes [25].

As a fundamental foundation of the healthcare system, electronic health records deliver data about a person’s health and medical care electronically and according to a common information model [26]. Healthcare professionals must have access to maternal immunization histories in electronic health records to ensure that the pregnant lady receives the proper shot and pertinent information [27].

The integrated health system (SIB) is one of the key sources of information for registering cases in the vaccination registry (case finding), taking into account the structure of the Iranian health system, the registration of information on households and pregnant women, the type of medical services, and the vaccination information for COVID-19 in this system [28].

Given the importance of vaccination of pregnant women, reducing the severity of COVID-19 infection in them and infants, as well as the need to record vaccination consequences, not possible without having a registry, establishing a national vaccine registry model to manage the pregnant women COVID-19 vaccination data is essential. Accordingly, this study aims to design a national registry model for COVID-19 vaccination of pregnant women as a policy to control complications of pregnant women’s vaccination in Iran.

## Methods

### Design and setting

**Fig. 1:**
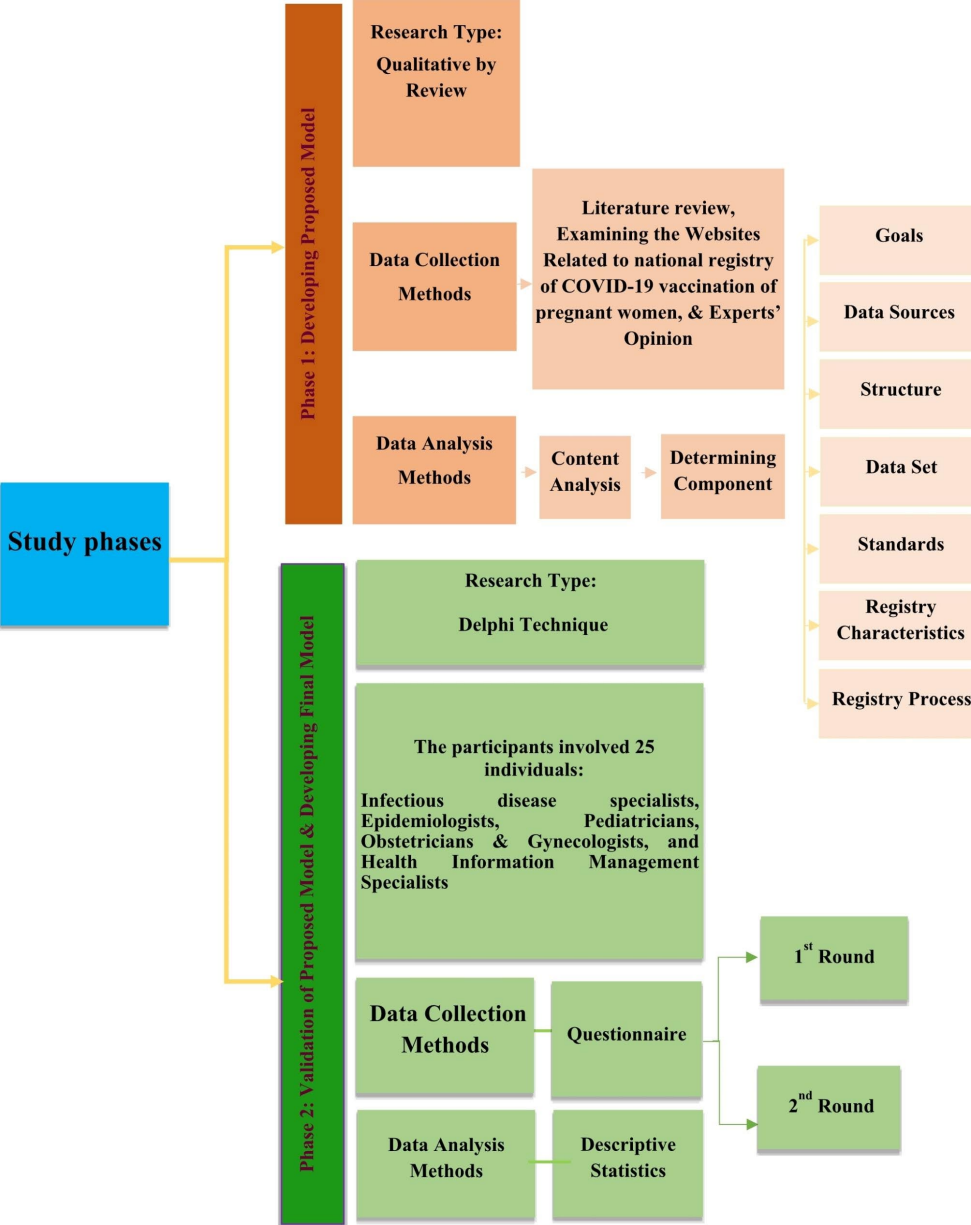
Final Abstract Graphic Flow Chart of the study

This research is an applied descriptive study conducted in Tehran, Iran, in 2021 and 2022 in two stages to design a national model of the COVID-19 vaccination registry for pregnant women in Iran. The study phases are shown in Fig. 1.

### Literature review

In the first stage, the coordinates of the National Registry of COVID-19 vaccination of pregnant women from related references and articles, as well as the comparative study of the National Registry of the COVID-19 vaccination of pregnant women in the United States, Canada, and the United Kingdom was done.

### Information sources and search strategy

Related articles published between 2019 and October 15, 2022, were extracted from PubMed, Springer, Science Direct databases, and Google Scholar search engine using a combination of keywords (MeSH terms) as well as useful websites and library resources were investigated. Table 1 presents the keywords used in the search to retrieve related articles.

**Table 1:** Search strategy in scientific databases

Time limitation	2019 to October 15, 2022
**Language limitation**	Only full text in English
**#1**	“COVID-19 Vaccines” OR “COVID-19 Virus Vaccines” OR “SARS CoV 2 Vaccines” OR “Coronavirus Disease 2019 Vaccines” OR “2019-nCoV Vaccines” OR “SARS Coronavirus 2 Vaccines”
**#2**	“Register*” OR “Data Management” OR “Information Management” OR “Surveillance System” OR “Data System” OR “Information System*”
**#3**	“Pregnant Women” OR “Pregnant” OR “Pregnancy”
**Search**	#1 AND #2 AND #3

### Eligibility criteria

#### Inclusion criteria

The criteria for inclusion in the study were original research articles that investigated The National Registry of COVID-19 vaccination of pregnant women in leading countries such as the United States, Canada and the United Kingdom.

#### Exclusion criteria

Articles that did not have enough details about the COVID-19 vaccination registry for pregnant women were excluded from the study. Non-authentic articles (e.g., review articles, editorials, & protocols) were excluded. Furthermore, articles with no full text (for any reason) were also excluded from this research.

### Study selection and data extraction

After retrieving the relevant articles, each article was independently reviewed by two authors (F A, R SH). Subsequently,, both authors provided the reason for the rejection of each article. In case of disagreement, other authors reviewed the article (R R, H E, A S).

After selecting the articles with inclusion criteria, the required data were collected using a data extraction form per the study’s objectives. The data extraction form consisted of five main parts: Objectives, data resources, participating organizations, minimum data set (MDS), and registry process. The content of this study phase was then analyzed, considering the aim of the study.

### Presenting the proposed model of the National Registry of COVID-19 vaccination in Iran

In the second stage, the preliminary model was designed based on objectives, data sources, data sets, data processing, reports, data quality control process, and patient follow-up.

### Validation of the proposed model and presentation of the final model

The model’s validity was established through content validity, based on reading reliable materials and gathering the opinions of subject-matter experts (using a two-stage Delphi technique). A questionnaire was created to validate the model. The responses to each question were “Agree” (a positive score) or “Disagree” (a negative score). A blank space was also provided beside each question for experts to express their reasons and/or suggest modifications. Test-retest and a correlation coefficient of 92% were used to determine its dependability. Using the expert sampling method [29], a questionnaire was given to faculty members of medical universities who were five infectious disease specialists, five epidemiologists, five pediatricians, five obstetricians and gynecologists, and five health information management specialists. The selection criteria for the panel members were sufficient expertise regarding the subject under review. Through the Delphi method, their opinions were gathered twice. A 22-item questionnaire about the axes of the proposed model was created and sent to the experts as part of the Delphi technique’s first stage. Based on the responses gleaned from the experts’ first-stage opinions, a 15-item questionnaire was created and distributed to the experts for the second stage of the Delphi technique. After analyzing the data, the final design of Iran’s national COVID-19 vaccine registry for expectant mothers was introduced.

The experts’ identities and responses were kept confidential during the registry of the COVID-19 vaccine in pregnant women model validation. Moreover, their participation in the validation stages was voluntary, and they were free to withdraw from the study at any stage.

## Results

The research findings are presented in three sections as follows:

Section 1: Findings from the comparative study of the COVID-19 vaccination registry of pregnant women in selected countries (Tables 2 and 3), Sect. 2: The proposed model of the national registry of COVID-19 vaccination in Iran (Table 4), and Sect. 3: Findings from the validation of the proposed model. (Table 5)

**Table 2:** Components of COVID-19 vaccination registry in pregnant women

Component / Country	United States	United Kingdom	Canada
**Objectives**	Establishing a supervision system to obtain more information about COVID-19 vaccination of pregnant women and their infants	Determining the characteristics of women who receive the COVID-19 vaccine during pregnancy, Determining and comparing the consequences of pregnancy for mothers and their infants.	Evaluation of immunity and efficacy of COVID-19 vaccines in pregnant women, Evaluation of attitudes toward COVID-19 vaccination in the population of pregnant women
**Data resources**	Obstetrics and gynecology clinics, pediatric clinics, Imaging centers, Genetics laboratories, Medical records	Obstetrics and teratology clinics	Obstetrics and gynecology clinics, Medical records
**Participating organizations**	Center for disease control and prevention, Epidemiological research center statistics center maternal, Fetal, and neonatal research centers for infectious diseases	National perinatal epidemiology unit, British teratology information service, Royal college of obstetricians and gynecologists, Medicines and Healthcare Products Regulatory Agency (MHRA)	Women’s health research institute, Vaccine evaluation center, Regional general hospital, Department of obstetrics and gynecology, Infectious diseases center, Women and children’s health association, COVID-19 immunity task force, Public Health Agency of Canada (PHAC)
**Minimum data set(MDS)**	Demographic information, Clinical data, COVID-19 vaccine information, Adverse events, Complications of pregnancy, Neonatal / infant outcomes	Demographic information, COVID-19 vaccine information, Complications of pregnancy, Neonatal / infant outcomes	Demographic information, Clinical data, COVID-19 vaccine information, Adverse events, Complications of pregnancy, Neonatal / infant outcomes

**Table 3:** COVID-19 vaccination registry processes in pregnant women

Process / Country	United States	United Kingdom	Canada
**Case finding**	Active	Active	Active
**Data collection**	Data collection tools: use of electronic report form. Responsible for data collection: CDC staff of International Organization for Standardization (ISO), Obstetricians, Pediatricians, Epidemiologists, Clinicians from the division of reproductive health and the division of birth defects and infant disorders, Trained registry experts	Data collection tools through electronic questionnaire form. Responsible for data collection: Gynecologists, Teratologists	Data collection tools through an electronic questionnaire
**Data quality control**	The review process includes reviewing all reported clinical data. The data is evaluated by several reviewers. Quality control criteria include a review in terms of consistency in case classification, quality of clinical review standards, and ensuring consensus in the interpretation of data and reported results	The content of data collection forms is thoroughly evaluated by the steering committee.	The data is evaluated by selected representatives of the principal investigator or the University of British Columbia (UBC) research ethics board for the purpose of supervises the research.
**Data processing**	Data processing is done using descriptive statistics.	Data processing is done using statistical indices (frequency and percentage).	Data processing is done using descriptive statistics
**Patient follow-up**	Active follow-up: Once every three months of pregnancy, Once and twice after childbirth, Once during delivery (in 4–8 weeks) and three months after childbirth, and up to 12 months after the final dose of the vaccine. Follow up is done by phone and via text message	Follow up every month How to follow up: By phone and Email, Post, Via text message	Short follow-up every two months after the first baseline survey How to follow up: By phone or Email
**Report**	Publication of reports related to the Advisory Committee on Immunization Practices (ACIP). 3-month reports in the form of updated articles and summaries	Publication of quarterly newsletters and annual reports	Publishing the reports as updated summaries via the website and email to the general public

**Table 4:** Proposed model of the national vaccine registry of COVID-19 in pregnant women

**Objectives**	$\checkmark$ Data collection from pregnant women and their infants to evaluate the immunity and efficacy of COVID-19 vaccines, $\checkmark$ Provide researchers and policymakers with information on how to use the best COVID-19 vaccine in pregnant women in the future		
**Data Resources**	Vaccination centers, Clinics (obstetrics, pediatrics, and genetics), Imaging centers, Integrated health system (SIB), and health information systems(HIS) in the relevant medical centers		
**Structure**	**Responsible organization**	Center for disease control and prevention at the ministry of health	
	**Registry Centers**	Urban centers of COVID-19 vaccination registry in pregnant women, Provincial centers of COVID-19 vaccination registry in pregnant women, National centers of COVID-19 vaccination registry in pregnant women	
	**Participating organizations**	National center for chronic disease prevention and health promotion, Epidemiological research center, Statistics center, Maternal, Fetal, and infant research centers, Women and children health association, Center for infectious diseases, National center for birth defects and developmental disabilities, National center for immunization and respiratory diseases, COVID-19 immunity task force, provincial / regional vaccination advisory committees	
	**Supervisory Committees**	Quality control committees of COVID-19 vaccination registry data of pregnant women, COVID-19 vaccination registry data disclosure committees for pregnant women, COVID-19 vaccination registry steering committee for pregnant women	
**The method of maintaining security and privacy**	$\checkmark$ Replacing the pregnant woman’s name with a unique code number so that the identity of the data is hidden, $\checkmark$ The required data and information should be encrypted and stored in digital format to ensure its security and confidentiality can be monitored by security standards, $\checkmark$ Only qualified and certified researchers and experts have access to the data		
**Data sets**	Demographic information, Clinical data, COVID-19 vaccine information, Adverse events of vaccination, Complications of pregnancy neonatal / infant Outcomes		
**Registry Processes**	**Inclusion and exclusion criteria**	Inclusion criteria: The population of pregnant women eligible for supervision through the pregnancy registry includes those exposed to COVID-19 vaccines during pregnancy (30 days before LMP (last menstrual period) to 14 days after LMP), Exclusion criteria: People who were not pregnant at the time of vaccination and were not pregnant for 30 days after vaccination	
	**Case finding method**	Active	
	**Case finding resources**	Cases reported by the pregnant woman or the health care provider, Integrated health system (SIB), Reports from health centers	
	**Data collection**	Based on the manual and electronic file reporting form by obstetricians, Pediatricians, Epidemiologists, Clinicians from the division of reproductive health and the division of birth defects and infant disorders, Trained registry experts	
	**Quality control**	**Quality control index**	Completeness, Definition, Timeliness, Accuracy of data, Validity, Non-duplication of data
		**Quality control methods**	Review of duplicates, Review of incorrect and missing information, Irrelevant and inappropriate, Audit of medical records, Review of the final report before each analysis
	**Data processing**	Frequency of COVID-19 vaccination in pregnant women, Frequency of local and systemic reactions reported on the day after vaccination, Calculation of post-vaccination adverse outcomes in pregnancy (including pregnancy complications, delivery results), Comparison of maternal and infants’ adverse outcomes in exposed and non-exposed groups of pregnant women	
	**Reports**	**Reporting method**	Providing periodic reports (monthly and annual), Updated articles and summaries, and general reports to provide information to pregnant women
		**Report users**	Researchers, Health care professionals, Health centers, Women’s health centers
	**Patients’ follow-up**	**Follow-up methods**	Phone contact and text message, Electronic communications (online)
		**Follow-up time intervals**	Monthly, Every three months (pregnancy), and in infants, once every three months until one year
**Standards**	**Classification systems and terminology**	ICD-10 3 ATC^4^ NDC^5^ LOINC^6^	
	**Nomenclature system**	SNOMED-CT^7^ MedDRA^8^	
	**Information exchange and messaging method**	HL7^9^	

**Table 5:** Frequency Distribution of Expert Opinions on the Proposed Model of COVID-19 Vaccination Registry in Pregnant Women for Iran (First Stage of Delphi Technique)

Expert Comments Components and registry processes	Agree		Disagree		Suggestions
	Number	Percent	Number	Percent	
**Objectives**	25	100	-	-	-
**Data resources**	20	80	5	20	The information on the desired items was recorded using the SIB system as one of the information sources
**Registry structure**	25	100	-	-	-
**Minimum data set**	25	100	-	-	-
**Standards**	20	80	5	20	It is proposed to use the latest standards in this field to develop registry software by changing standards and advancing technology
**Registry processes**	21	84	4	16	The ministry of health is considered one of the users of registry reports, A dynamic report maker should be added to the collection in software development based on the proposed model.

The study’s findings indicate that the countries that have established a national registry of COVID-19 vaccination have similar objectives for monitoring the COVID-19 vaccination process in pregnant women. Tables 2 and 3 summarize the results of the comparative registry study in selected countries. These tables provide the coordinates of the national COVID-19 vaccine registry for pregnant women and the main registry processes.

Part 1: Findings from a comparative study of the COVID-19 vaccination registry in pregnant women in selected countries.

Part 2: Proposed model of the national vaccine registry for COVID-19 in pregnant women in Iran.

Part 3: Validation of the Proposed Model for the National COVID-19 Vaccination Registry in Pregnant Women.

In the second stage of the Delphi approach, after considering the first stage suggestions and forming an expert panel, all components were approved by 100% of the experts.

## Discussion

Vaccines are the most effective way to prevent contagious diseases and reduce morbidity and mortality rates without long-term side effects [30]. COVID-19 vaccination is immune and effective in preventing the severe consequences of COVID-19 infection, including death. The American College of Obstetricians and Gynecologists(ACOG) and the Society of Maternal and Fetal Medicine (SMFM), two leading obstetric care organizations, recommend that all pregnant women be vaccinated against COVID-19 [16]. The information related to vaccine exposure during pregnancy is vital to monitor the immunity and effectiveness of vaccines. Therefore, establishing a National Registry of COVID-19 pregnancy vaccines can be a significant step toward collecting, analyzing, and distributing vaccination data and its complications [31, 32]. The COVID-19 vaccination in pregnant women is recommended when the benefits of vaccination for pregnant women outweigh the potential risks. To assist pregnant women in performing this assessment, they should be provided with information on the risks of COVID-19 in pregnancy, the potential benefits of vaccination in the epidemiological context, and the current limitations of immunity data in pregnant women [33–36]. Determining the goals is one of the first steps in planning a registry [22]. In this study, the objectives of the national COVID-19 vaccine registry in pregnant women include collecting data from pregnant women and their infants to evaluate the immunity and efficacy of COVID-19 vaccines, giving information to the researchers and policymakers on how to use the best COVID-19 vaccine in pregnant women and the future.

The most critical processes of the registries consist of case finding, data collection and storage, data summarization, patient follow-up, reporting, and data quality control [37–39]. The present study’s designed national model of pregnant COVID-19 vaccination includes the above processes.

An applied method for case finding is active and automatic communication of health centers [22], done actively in the present study. The proposed model’s data sources include vaccination facilities, offices and clinics (for gynecology, obstetrics, pediatrics, and genetics), imaging facilities, integrated health systems, and health information systems in the relevant medical centers. These sources were chosen based on research in a few selected countries and the country’s health system structure. Additionally, the following inclusion criteria used in the registry include during pregnancy or the gestation period (30 days before LMP to 14 days after LMP), women were exposed to the COVID-19 vaccination. Exclusion criteria include people who were not pregnant at the time of vaccination or who will not become pregnant until 30 days after vaccination and will not be included in the registry.

V-safe Surveillance System and Pregnancy Registry is a new smartphone-based active surveillance system developed for the COVID-19 vaccination program. The registry is administered by the CDC and the Food and Drug Administration (FDA) in the USA. Case finding in this registry is done through healthcareproviders. Healthcare providers are required to report certain adverse events after vaccination, including pregnancy-related complications resulting in hospitalization and congenital anomalies, for Covid-19 vaccines [40].

A minimum data set is a common data set that should be used to collect data in a registry [41, 42]. The minimum dataset serves as a standard tool for collecting data, essential for providing precise and reliable healthcare services [43]. In addition, it is valuable in improving data quality and is practical for planning, developing, monitoring, managing, and evaluating performance. In addition, the minimum data set increases the accuracy and comprehensiveness of information and ultimately leads to the presentation of high-quality healthcare [42, 44, 45]. Therefore, this study’s minimum data set includes demographic information, clinical data, COVID-19 vaccine information, adverse events, pregnancy complications, and Neonatal / Infant outcomes.

The minimum data set of Pregistry International Pregnancy Exposure Registry (PIPER) includes information on maternal and infant medical conditions, new COVID-19 vaccination doses, use of medications, environmental exposures, and results of SARS-CoV-2 tests collected during pregnancy and until 12 months after delivery for all live births [46].

Vaccination immunity assessment in pregnant women requires more precautions to properly monitor pregnancy and neonatal consequences. Awareness of the background of adverse pregnancy and neonatal consequences among the study population is also needed to accurately assess causation [47]. Active monitoring of vaccination immunity is recommended in addition to inactive reporting systems because, currently, insufficient information is available on the safety of the COVID-19 vaccine in pregnant women. Active monitoring involves collecting, analyzing, and interpreting data. Besides, active monitoring aims to identify ongoing adverse events in pregnant women and their children. Identified events can be used to determine the extent of specific adverse events and identify any trends or changes through a continuous pre-organized process in this group.

In the pregnancy exposure registries (PERs), the rate of these events can be compared to cases that have not been exposed to a concurrent or historical group of pregnant women, which facilitates the assessment of the risk associated with vaccination. Collecting comparable data in different applications is essential to enable data coordination and comparison [19]. The present study’s data collection is based on manual and electronic file reporting forms by obstetricians, pediatricians, epidemiologists, reproductive health physicians, physicians of disabilities congenital and neonatal disorders unit, and trained registry experts.

In general, using standard forms for collecting data and abstracting increases the data quality, primarily in terms of its comprehensiveness and precision [48]. In order to maximize the people’s participation in the pregnancy exposure registries, it is recommended to employ new technologies for gathering data.

Training should be provided to improve clinical records and determine outcomes using standardized case definitions. All living and dead infants should be examined and weighed, and any congenital abnormalities should be identified and referred to a specialist for examination. Expected adverse birth consequences such as low birth weight, preterm birth, and small gestational age and their rates for comparison between groups to identify any differences should be documented. Therefore, PERs can assess data quality, describe the epidemiology of exposure and group outcomes, and determine and compare event rates [19]. In the proposed model, data processing includes the percentage of the frequency of COVID-19 vaccination in pregnant women, the percentage of the frequency of local and systemic reactions reported the day after vaccination, the calculation of the likelihood of adverse pregnancy outcomes following vaccination (including pregnancy complications and delivery outcomes), and a comparison of the ratio of adverse maternal and infant outcomes in exposed and non-exposed groups of pregnant women. In order to ensure the information’s quality and security, it is also advised that control and disclosure committees be established in the registry.

In the proposed model, data quality indices include completeness, definition, and timeliness, the accuracy of data, validity, non-duplication of data, as well as quality control methods, including checking duplicates, checking incorrect and missing information, irrelevant and inappropriate, auditing medical records, and checking the final report before each analysis. Establishing control committees and disclosing information in the registry regarding the quality and security of information is also recommended.

Patient follow-up is a crucial function in the registry to evaluate treatment outcomes [49]. In this study, pregnant women are followed up monthly, and newborns are followed up once every three months until age one. Reporting is another essential characteristic of registries [50]. Identifying indices and reporting through different methods allows data to be compared at different levels of decision-making [12, 51]. Accordingly, various reports and reporting methods are proposed in the presented model.

One of the limitations of the C-VIPER registry is that some adverse outcomes, such as neurodevelopmental delay and some major congenital malformations, may not become apparent until after 12 months of age and may therefore be missed [24]. In order to follow up and enhance people’s involvement, incentives are necessary.

Considering that one of the priorities of the research and technology department of the Ministry of Health and Medical Education is to set up registry systems for various diseases and procedures, the model presented in this study can create a suitable information platform for the design and implementation of the COVID-19 vaccination registry in pregnant women. Considering that the prevalence of the acceptance of the COVID-19 vaccine in pregnant women is lower than the general vaccination of COVID-19, which can be due to the lack of sufficient knowledge and awareness [52], it is suggested that the necessary interventions to increase the acceptance of the vaccine, address safety concerns and education in to be done.

## Conclusion

Registries are one of the most crucial evaluation tools for data management, so a national COVID-19 vaccination registry for pregnant women helps manage COVID-19 vaccination information during pregnancy. Accurate and standard execution of such a registry must consider a comprehensive model that evaluates different design dimensions.

## Data Availability

All data generated or analyzed during this study are included in this published article. All authors read and approved the final version of the manuscript, had full access to all of the data, and took complete responsibility for the data’s integrity and the data analysis’s accuracy. The data supporting this study’s findings are available through the corresponding author, [Farkhondeh Asadi], upon reasonable request.

## Footnotes

1 Preeclampsia (a serious blood pressure disorder).

2 Coagulopathy (a blood clotting disorder).

3 International Statistical Classification of Diseases and Related Health Problems(ICD-10).

4 Anatomical Therapeutic Chemical Classification (ATC).

5 National Drug Code(NDC).

6 Logical Observation Identifiers Names and Codes(LOINC).

7 Systemized Nomenclature of Medicine – Clinical Terms.(SNOMED-CT).

8 Medical Dictionary for Regulatory Activities Terminology (MedDRA).

9 Health Level 7(HL-7).

## Abbreviations

ARDS: Acute Respiratory Distress Syndrome
NICU: Newborn Intensive Care Unit
CDC: Centers for Disease Control and Prevention
SMFM: Society for Maternal and Fetal Medicine
VPDs: Vaccine-Preventable Diseases
VAERS: Vaccine Adverse Event Reporting System
EHRs: Electronic Health Records
NCVHS: National Committee on Vital and Health Statistics
COVIPER: COVID-19 Vaccines International Pregnancy Exposure
MHRA: Medicines and Healthcare Products Regulatory Agency
PHAC: Public Health Agency of Canada
MDS: Minimum Data Set
ISO: International Organization for Standardization
UBC: University of British Columbia
ACIP: Advisory Committee on Immunization Practices
LMP: Last Menstrual Period
ICD-10: International Statistical Classification of Diseases and Related Health Problems
ATC: Anatomical Therapeutic Chemical Classification
NDC: National Drug Code
LOINC: Logical Observation Identifiers Names and Codes
SNOMED-CT: Systemized Nomenclature of Medicine Clinical Terms
MedDRA: Medical Dictionary for Regulatory Activities Terminology
HL-7: Health Level 7
ACOG: American College of Obstetricians and Gynecologists
PERs: Pregnancy Exposure Registries
FDA: Food and Drug Administration
